# Antibiotic growth promoter and phytogenic feed additive consistently alter microbial community structure in chicken cecum

**DOI:** 10.3389/fmicb.2026.1702973

**Published:** 2026-06-04

**Authors:** Chengyao Peng, Giorgia delle Grazie, Mahdi Ghanbari, Ali May, Thomas Abeel

**Affiliations:** 1 Delft Bioinformatics Lab, Delft University of Technology, Delft, Netherlands; 2 Department of Mathematical Sciences, Polytechnic University of Turin, Torino, Italy; 3 dsm-firmenich, Animal Nutrition and Health R&D Center, Tulln, Austria; 4 dsm-firmenich, Science & Research, Delft, Netherlands; 5 Infectious Disease and Microbiome Program, Broad Institute of MIT and Harvard, Cambridge, MA, United States

**Keywords:** antibiotic growth promoters (AGPs), broiler chicken, comparative network analysis, co-occurrence network, microbiome, phytogenic feed additives (PFAs)

## Abstract

**Background:**

Efforts to replace antibiotic growth promoters (AGPs) in livestock are often hindered by a limited mechanistic understanding of how sub-therapeutic antibiotic doses enhance animal growth. Since AGP concentrations are typically too low to directly suppress pathogens, their effects on the gut microbiome, particularly its ecological dynamics, warrant closer investigation. A critical but underexplored dimension is how these additives influence the structure and stability of microbial communities as interconnected ecosystems.

**Methods:**

We conducted a comparative network-based analysis to examine the effects of zinc-bactracin, a commonly used AGP, and Digestarom^®^, an alternative phytogenic feed additive (PFA) on cecal microbiome dynamics in broiler chickens. Using metagenomic data from a repeated cross-sectional randomized controlled trial of 96 broiler chickens assigned to three dietary groups: Basal (Control), AGP and PFA, we constructed microbial co-occurrence networks using Spearman's correlation for birds raised on basal, AGP-, or PFA-supplemented diets at key developmental stages (Day 3, 14, 21, and 35). We assessed changes in network topology, modular organization and node centrality. We evaluated whether the network-prioritized keystone taxa could discriminate among diets using a Random Forest classifier.

**Results:**

Compared to the Control group, both AGP and PFA treatments induced consistent shifts in network topology, including reduced connectivity, increased modularity, increased percentage of positive interactions, enhanced mucosa connectivity, and improved structural robustness over experiment time. Overall, these treatment-induced changes were more pronounced under AGP than under PFA. Despite these changes, we identified conserved subgraphs with stable interconnections across diets and time points during the experiment. The node centrality analysis revealed condition-specific keystone taxa, but Linear Discriminant Analysis (LDA) and Random Forest (RF) struggled to accurately differentiate between diets using their abundance, particularly between PFA and the two other groups.

**Conclusion:**

Our findings reveal that feed additives can reshape gut microbial dynamics without producing marked compositional shifts. The consistent network-level changes observed for both AGP and PFA highlight the value of ecological network analysis in uncovering microbial community responses. These insights improve our understanding of cecal microbiome responses in chickens, highlight potential modes of action of AGPs, and offer a comparative framework for assessing the microbial impacts of alternative feed additives.

## 1 Introduction

Over the past few decades, chicken farming has achieved remarkable gains in production efficiency, largely driven by genetic selection and optimized nutrition. Among the contributing factors, the inclusion of antibiotics in feed—commonly known as antibiotic growth promoters (AGPs)—has historically played a significant role in promoting growth and improving feed efficiency in broiler chickens (Zou et al., 2022). However, the widespread use of antibiotics in animal agriculture has raised serious public health concerns due to its link with the emergence and spread of antimicrobial resistance (AMR) in both animals and humans (Khachatourians, 1998; Singer et al., 2003). In response, regulatory measures have been introduced worldwide, including a complete ban on AGPs in the European Union since 2003 (Casewell et al., 2003). These restrictions have prompted the livestock industry to search for viable alternatives that maintain animal health and productivity without contributing to AMR. Phytogenic feed additives (PFAs), plant-derived compounds with antimicrobial, antioxidant, and digestive-modulating properties, have emerged as a promising alternative to AGPs (Murugesan et al., 2015).

Despite their potential, the development and validation of effective alternatives have proven challenging. This is partly because the growth-promoting effects of AGPs are not solely attributable to pathogen suppression but may also involve more complex alterations in gut microbiota composition and function. Recent advances in high-throughput sequencing have enabled more detailed investigation of how AGPs affect the gut microbiome across various livestock species, including poultry (Lin et al., 2013; Costa et al., 2017; Oladokun et al., 2022; Paul et al., 2022). Most of these studies have focused on macro-level descriptors such as microbial diversity, richness, or the change in the abundance of specific taxa to characterize the impact of feed additives on the animal gut microbiome. While informative, such approaches often overlook the ecological complexity of the gut microbiome, where hundreds of microbial species interact dynamically in a densely populated environment. An ecological lens, incorporating concepts like stability, resilience, and community assembly, may offer deeper insights into the functional consequences of microbiome perturbations (Ley et al., 2006; Johnson and Burnet, 2016). Co-occurrence networks, for instance, integrate these ecological concepts and allow such investigation of animal gut microbiome, including the ones from chicken (Feng et al., 2023; Huang et al., 2018; Wen et al., 2021). As the name implies, these networks are constructed based on the co-occurrence patterns in the microbiome data, with taxa as nodes and their associations as edges. Network properties, such as modularity, robustness, and keystone taxa, can be used to characterize the microbiome community, with a particular focus on its stability (Kajihara and Hynson, 2024). Since gut microbiota stability is considered essential for host health and well-being, examining how the feed additives, such as AGPs, influence the stability of the animal gut microbiome community may be key to elucidating their grow-promoting mechanisms. However, systematic co-occurrence network analyses that examine detailed network properties remain scarce in microbiome research and are particularly underexplored in the context of feed additive use in livestock production.

In this study, we comprehensively investigated how dietary inclusion of an antibiotic at growth-promoting levels and a phytogenic feed additive influences the cecal microbiota of broiler chickens, conceptualized as a dynamic and interconnected community. The cecum, a key site for microbial fermentation and nutrient salvage in chicken, was selected as the focal point for assessing microbial responses. Leveraging data from a previously published randomized controlled trial (Segura-Wang et al., 2021), we constructed microbial co-occurrence networks from cecal metagenomic profiles of chicken at different developmental stages, raised on either a basal diet, a basal diet supplemented with a common AGP (zinc-bacitracin), or a basal diet supplemented with a commercial phytogenic feed additive (Digestarom^®^). These networks capture shifts in the microbial community structure and interaction patterns shaped by the different dietary interventions. We then applied a comprehensive suit of network analyses, including topological characterization, virtual disturbance simulations, and node centrality assessment, to uncover consistent alterations in microbial community organization induced by the two feed additives. Through this ecological lens, we aim to identify feed additive-induced shifts in gut microbiome dynamics that conventional taxonomic analyses may overlook and to generate new mechanistic insights relevant to the development of sustainable feed strategies in post-antibiotic livestock systems.

## 2 Materials and methods

### 2.1 Materials

The data used in this study was from a metagenomic dataset previously published by Segura-Wang et al. (2021) (BioProject accession PRJNA715658 in NCBI), with the experimental details described by Peng et al. (2024). Briefly, 96 one-day-old healthy chickens were randomly assigned to twelve pens, with eight chickens per pen. These pens were later randomized into three treatment groups: Control(CTR), AGP and PFA, with four replicate pens per group. All birds were fed with a standardized basal diet throughout the experiment. In addition to the basal feed, chickens in the AGP group received supplementation with a commonly used antibiotic administered for growth promotion, zinc-bactracin (ALBAC^®^, Huvepharma, Belgium; 20 mg/kg), while those in the PFA group were supplemented with a commercial PFA (Digestarom^®^ DC Power, Biomin Holding GmbH, Austria; 150 mg/kg feed). Cecal metagenomic samples from digesta and mucosa regions were collected at four time points (days 3, 14, 21 and 35), with four replicates per treatment group at each time point. The samples from day 3 were collected just before the treatment, serving as baselines.

### 2.2 Quality control and taxonomic profiling of the metagenomic dataset

Quality control of metagenomic data was performed using FastQC v0.11.7 (Andrews et al., 2017), followed by adapter removal and quality trimming with Trimmomatic v0.39 (Bolger et al., 2014). Host DNA contamination was eliminated by removing reads that mapped to the chicken reference genome (Gallus gallus GRCg6a, obtained from NCBI at https://ftp.ncbi.nlm.nih.gov/genomes/all/GCF/000/002/315/GCF_000002315.6_GRCg6a/. The read alignment was performed using Bowtie2 v2.3.5.1 (Langmead and Salzberg, 2012) and read removal using samtools v1.10 (Li et al., 2009).

Taxonomic classification and abundance profiling were conducted using Kraken2 v2.1.2 (Wood et al., 2019) with the GTDB release 207 database (Parks et al., 2022) prepared via Struo2 v2.3.0 (Youngblut and Ley, 2021). The taxonomic assignments were refined using Bracken v2.9 (Lu et al., 2017). Low-abundance taxa were filtered from downstream analyses by removing microbial species with relative abundances less than 0.01% present in fewer than 10% of samples to minimize technical artifacts. As in the rest of the method section, the scripts and analysis were implemented under Python v3.11.

### 2.3 Co-occurrence network construction

To alleviate spurious correlations as a result of compositionality in the data, we applied the Centered Log-Ratio (CLR) transformation to the species-level abundance obtained from the previous step. Subsequently, we constructed co-occurrence networks for the three diet groups at four time points with the corresponding eight replicates by a correlation-based method. We chose to combine the replicates from the two sample types, digesta and mucosa, due to the very high positive correlation identified by Mantel test in the original paper (Peng et al., 2024). To prevent indirect correlations resulting from the sample type variable, we added the two sample types as virtual nodes into the networks. Therefore, at each of the four time points, eight metagenomic samples were used to infer the co-occurrence network for each of the treatment groups (CTR, AGP and PFA).

Specifically, we used Spearman rank correlation (from SciPy v1.16.2) and applied permutation and bootstrap procedures to construct sparse networks. In detail, we shuffled taxon abundances and resampled with replacement across 1,000 iterations to generate permutation and bootstrap distributions. Then, a Gaussian curve was fitted to the bootstrap distribution to estimate *null* value probabilities. To address compositionality bias, we included a renormalization step during the permutation process. Finally, we applied multi-testing correction using the Benjamini-Hochberg method (Benjamini and Yekutieli, 2001) (from statsmodels v0.14.4) to adjust *p*-values and only the correlations with adjusted p-values below 0.05 were retained as edges in the final networks.

### 2.4 Comparative network analysis

#### 2.4.1 Topological structure and organization

At each time point, we compared the constructed co-occurrence networks by calculating various topological characteristics. These include network metrics, such as the number of nodes, edges, components, percentage of positive edges, mean degree, the size of the largest connected component, as well as community structure properties, such as modularity and mean clustering coefficient. For modularity calculation, we used Python package community v0.16 and for the other metrics, we utilized networkx v3.5. To confirm that the resulting networks are not due to chance, we performed a z-test, implemented in statsmodels v0.14.4, to compare the topological measures of the observed network with those from 100 random networks, which maintained the same degree distribution as the observed network. The resulting networks were visualized by Gephi v0.10.1 (Bastian et al., 2009). A detailed description of mean degree, largest connected component (LCC) size, modularity and mean cluster coefficient is provided below.

**Mean degree**: measures the average number of edges a node has in a network, which characterizes the overall connectivity network density. Since our networks are undirected, it was calculated using the following formula.


$$\begin{array}{l} k_{avg}=\frac{2E}{N} \end{array}$$ (1)

Where *E* is the total number of edges in the graph and *N* is the total number of nodes in the network. The mean degree indicates the complexity of the microbial interactions in the network.

**The Largest Connected Component (LCC) size**: measures the fraction of the network nodes that belong to the largest connected component (LCC) of a network, the largest subgraph of the network, in which every pair of nodes is connected by at least one path.

**Modularity**: measures the extent to which the network can be divided into densely connected sub-networks. We used the Louvain algorithm Baldassano and Bassett (2016a) to calculate the modularity, a hierarchical method that refines the partition of the network. This method divides the nodes into separate, non-overlapping groups to maximize the modularity index *Q*, which represents the quality of the network's division into distinct modules. The *Q* is computed with the following formula.


$$\begin{array}{l} Q=\frac{1}{2A}\underset{i}{\sum }\underset{j}{\sum }\left(A_{ij}-\gamma \frac{A_{i}A_{j}}{2A}*\delta \left(C_{i},C_{j}\right)\right) \end{array}$$ (2)

Where *A*_*ij*_ is the adjacency matrix, *A*_*i*_ is the degree of the node *i*, *A* is the average degree of the network, *C*_*i*_ is the community to which node *i* is assigned, and γ is a scaling parameter used to adjust the size of the modules. A higher *Q* value indicates a stronger modular structure, reflecting a clearer separation of the network into groups. Since multiple network configurations can produce a maximum *Q*, we carried out modularity optimization 1000 times to create a consensus partition. This consensus is formed by merging several partitions, assigning each node to the community it most often belongs to across all iterations. Modularity identification helps us find groups of related microbes.

**Mean clustering coefficient**: measures how interconnected an average node's neighbors are to each other in the network. For any given node *i* within a network, the clustering coefficient *CC*(*i*) is defined as followed.


$$\begin{array}{r} CC\left(i\right)=\frac{1}{N}\overset{N}{\underset{i=1}{\sum }}\frac{\text{number of triangles connected to }i}{\text{number of possible triangles connected to }i}\\ =\frac{1}{N}\overset{N}{\underset{i=1}{\sum }}\frac{2E_{i}}{k_{i}\left(k_{i}-1\right)} \end{array}$$ (3)

Where *E*_*i*_ is the number of triangles centered in node *i*, *k*_*i*_ is the degree of that node, and *N* is the total number of nodes. In simple words, the clustering coefficient measures how strongly a node's neighbors are connected to each other. To represent the overall connectivity strength of the network, we take the average of the clustering coefficients across all nodes.

#### 2.4.2 Robustness of microbial networks

We conducted node deletion experiments in two scenarios to quantify the influence of the use of feed additives on the stability and robustness of the microbial interaction network. In the first scenario, nodes were randomly removed from each network in steps of 5 nodes, approximately 1% of the total nodes, which was repeated over 100 independent iterations. In the second scenario, nodes were removed in the same stepwise manner, but in descending order of their influence within the network. To quantify this influence, we adapted a method proposed by Berry and Widder (2014), which demonstrated that the topological features of the microbial network, specifically the centrality of the node and the clustering coefficient, can effectively identify keystone taxa with high precision. Specifically, the node influence (*NI*) was calculated using its degree centrality, closeness centrality, betweenness centrality, and clustering coefficient. These measures were normalized using min-max normalization to account for their different ranges, so no single metric can disproportionately influence the influence score. In essence, we prioritized the species that are highly connected both locally and globally within the network, excluding the ones serving as connectors between the clusters.


$$\begin{array}{l} \text{NI}=\sum (\bar{\text{Degree Centrality}},\bar{\text{Closeness Centrality}},\\ \bar{\text{Clustering Coefficient}})-\bar{\text{Betweenness Centrality}} \end{array}$$ (4)

At each step, the robustness of the networks was quantified by the remaining edge fraction, LCC size fraction, and natural connectivity. Since natural connectivity can be strongly affected by the network, we normalized the natural connectivity at each step by dividing it by the original natural connectivity to reflect the change in natural connectivity that is independent of the network size.

#### 2.4.3 Core association identification

We used anuran package v1.1.0 (Rttjers et al., 2021) to investigate conserved microbial associations between constructed networks to identify the core microbial interactions. In short, *anuran* detects conserved network structures by comparing the observed networks with a null model generated through network shuffling, allowing for statistical identification of significantly shared edges.

### 2.5 Classifying feeding groups based on keystone taxa

We defined keystone taxa as the 50 most influential nodes in a given network based on the centrality of the nodes (10% of the total nodes), prioritized using the same formula aforementioned (4). To test whether those taxa can characterize the microbial communities during the experiment under different feeding regimes, we applied both a linear (Linear Discriminant Analysis) and a non-linear classifier (Random Forest). From the nine networks constructed during the treatment, we obtained nine distinct groups of keystone taxa from each network, one for each combination of treatment and age group. Subsequently, to get a single set of keystone taxa for each treatment, we merged the sets over the experiment time (day 14, 21 and 35) for each diet group. The CLR-transformed abundance of these keystone species, gut section and the development age were used to distinguish between the treatment groups. For LDA, abundance features were also standardized using z-score standardization.

Both classifiers were evaluated using the same nested cross-validation with an outer loop of 5-fold stratified cross-validation, which was repeated 10 times to robustly evaluate model performance. Within each outer fold, mutual information was used to select the top 15 features to reduce dimensionality. The inner loop employed 5-fold stratified cross-validation to optimize classifier hyperparameters to maximize classification accuracy. Both LDA and RF hyperparameters were tuned through Bayesian optimization provided in Optuna (v4.4.0) with 30 trials per inner fold, with their names and their ranges detailed in Supplementary Table 1. Model performance was evaluated on each outer test fold using accuracy and macro F1. We also used per-class F1 score and confusion matrix to obtain a more detailed assessment of classification performance across all groups. To establish a simple baseline, we trained a stratified dummy classifier to assess whether the optimized classifiers are learning meaningful patterns from the keystone taxa beyond those expected by chance.

## 3 Results

### 3.1 Feed additives increased microbial network sparsity, modularity and altered interaction balance

To investigate the response of the microbial community to AGP and its natural alternative, we used a correlation-based method to build co-occurrence networks of cecum microbiome at the species-level and performed a comparative network analysis. In total, twelve networks were constructed that represent the microbial dynamics in four developmental stages of chickens. The three networks constructed at day 3 were used as baselines. After the start of the treatment, three networks were constructed at the following experimental time points - day 14, day 21 and day 35—using metagenomic samples collected under basal diet or diets supplemented with an AGP or a PFA. Each network consists of the profiled microbial species as nodes and statistically significant associations as edges, reflecting microbial interactions within a specific dietary and temporal context.

As shown in Table 1, on day 3, the baseline time, the microbial networks built for three groups shared similar topological characteristics, indicating that the initial microbial community structures were comparable across groups before the start of the experiment. Following the intervention (on day 14, 21, and 35), however, although the two feed additive groups maintained a similar number of species (nodes) compared with the Control group, they consistently displayed increased network sparsity, with fewer network connections and lower mean degree. For example, on day 35, all groups had a comparable number of nodes, yet the CTR network had nearly twice the number of edges and a mean degree of 136.7, compared to 86.2 in the PFA network and 78.9 in the AGP network. Despite reduced connectivity, both feed additive networks exhibited markedly higher modularity across all post-treatment time points. Again on day 35, for example, the Control network had much lower modularity (0.24) than PFA (0.54) and the AGP (0.62) networks. To ensure that the observed community topological metrics (modularity and clustering coefficient) were not due to chance, we constructed random networks with the same number of nodes, edges, and degree distribution. The comparisons using z-tests confirmed the statistical significance of both metrics across all generated networks, with the significance level *p* < 0.05.

**Table 1:** Topological characteristics of the nine constructed microbial co-occurrence networks under different dietary conditions across the experiment time.

Age	Group	Nodes	Edges	Pos. edge %	Mean deg	Comp.	LCC	Modularity	Clust. coeff.
Day 3	CTR	520	32193	0.99	123.8	5	0.91	0.29	0.97
	PFA	524	36416	0.99	139	7	0.92	0.35	0.97
	AGP	517	33482	0.99	129.5	6	0.94	0.29	0.96
Day 14	CTR	503	16798	0.94	66.8	4	0.94	0.45	0.83
	PFA	510	14697	0.93	57.6	4	0.93	0.61	0.84
	AGP	496	13168	0.88	53.1	2	0.98	0.67	0.83
Day 21	CTR	511	32201	0.93	126.0	1	1	0.21	0.78
	PFA	513	20337	0.95	79.3	2	0.99	0.54	0.85
	AGP	520	16657	0.89	64.1	5	0.99	0.69	0.85
Day 35	CTR	531	36300	0.78	136.7	1	1	0.24	0.74
	PFA	517	22287	0.93	86.2	3	0.98	0.54	0.73
	AGP	520	20523	0.94	78.9	4	0.99	0.62	0.80

Each row in the table corresponds to a network built from species-level taxonomic profiles of eight biological replicates. The networks are grouped by the developmental age and treatment group (CTR, Control; PFA, Phytogenic Feed Additive; AGP, Antibiotic Growth Promoter), with their corresponding topological characteristics listed in each row. Pos. edge % denotes the percentage of positive edges; Mean deg indicates the mean degree; Comp. represents the number of components; LCC denotes the size of the largest connected component; and Clust. coeff. refers to the clustering coefficient.

In addition to structural differences, we observed changes in the balance between positive and negative associations across treatments and over time. At day 3, all networks were dominated by positive associations ( 99%), reflecting highly cooperative or co-varying microbial communities at this early developmental time. As the microbiome matured, the proportion of positive edges declined markedly in the Control group between day 21 and day 35 (from 0.93 to 0.78), whereas it remained at a consistently high level in the feed additive-supplemented groups (from 0.95 to 0.93 for PFA and from 0.89 to 0.94 for AGP).

We also noticed a time-dependent effect from both feed additives, with increased network sparsity, modularity, and a sustained predominance of positive associations becoming more pronounced at later time points. Meanwhile, the large connected component size and mean clustering coefficient remained quite stable across the dietary groups and time points.

### 3.2 Feed additives increase robustness of the microbial network structure in disruptions targeted at influential taxa

To assess whether the topological changes associated with the use of feed additives alter the overall network robustness, we performed node deletion experiments and quantified the remaining edge fraction and natural connectivity of the resulting networks over the experiment period. Our experiments revealed that both feed additives, especially AGP, increased the network robustness in node removal experiments targeted at influential nodes, which were prioritized using the same methods for keystone taxa identification.

**Figure 1:**
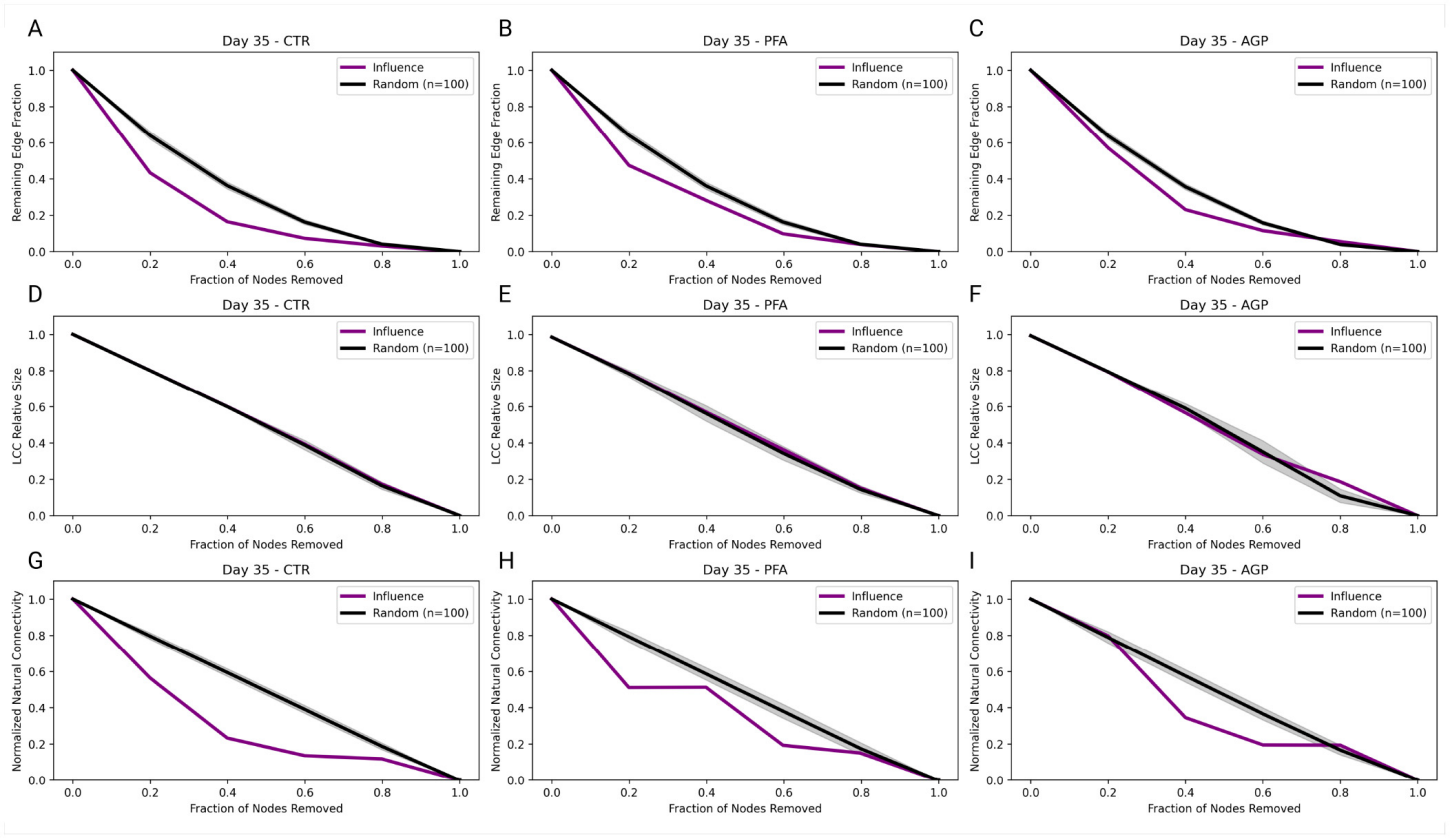
Remaining edge, LCC size and natural connectivity fraction during random and influential node removal experiments on microbial networks at day 35. Node removal was performed in around 1% increments (5 nodes), in a random order or based on node influence scores. Panels show the changes in remaining edge fraction in **(A–C)**, the remaining LCC size fraction in **(D–F)**, and normalized natural connectivity in **(G–I)** for the CTR, PFA and AGP networks, respectively. Random node removal experiments were repeated 100 times and the plotted values represent the mean ± standard deviation.

As shown in Figure 1, during influence-based node removal, networks from feed additive-supplemented samples (Figures 1B, C) experienced slightly less abrupt loss in remaining connections compared to the Control network samples (Figure 1A) at day 35. Meanwhile, microbial networks supplemented with feed additives exhibited more gradual decreases in the remained natural connectivity during influential node removal experiments (Figures 1G–I). In contrast, no effects has been established with the size of the remaining largest connected component (LCC) (Figures 1D–F). Notably, this increased robustness associated with feed additives indicated by the remaining edge fraction and natural connectivity was also observed at the other two experimental time points, day 14 and day 21 (Supplementary Figure S1).

To better understand the results, we plotted the degree distribution of the nodes of each of these networks in the Supplementary Figure S2. As shown, during the experiment, the Control networks consistently exhibit a bimodal degree distribution, with two widely separated peaks. In other words, the species in this case tend to fall into either of the two categories: those that are weakly connected with the other species and those that serve as strong hubs in the networks. In contrast, the degree distributions of additive-supplemented feed networks are narrower and tend to have more peaks. This structural difference suggests that the feed additive networks preserve overall connectivity more effectively during influential node removal experiments because of their less centralized topology.

In general, these consistent findings suggest that food additive supplementation increases the resilience of the network to disruptions targeted at crucial taxa in the community.

### 3.3 Feed additives promote mucosal colonization of species from a conserved network module

**Figure 2:**
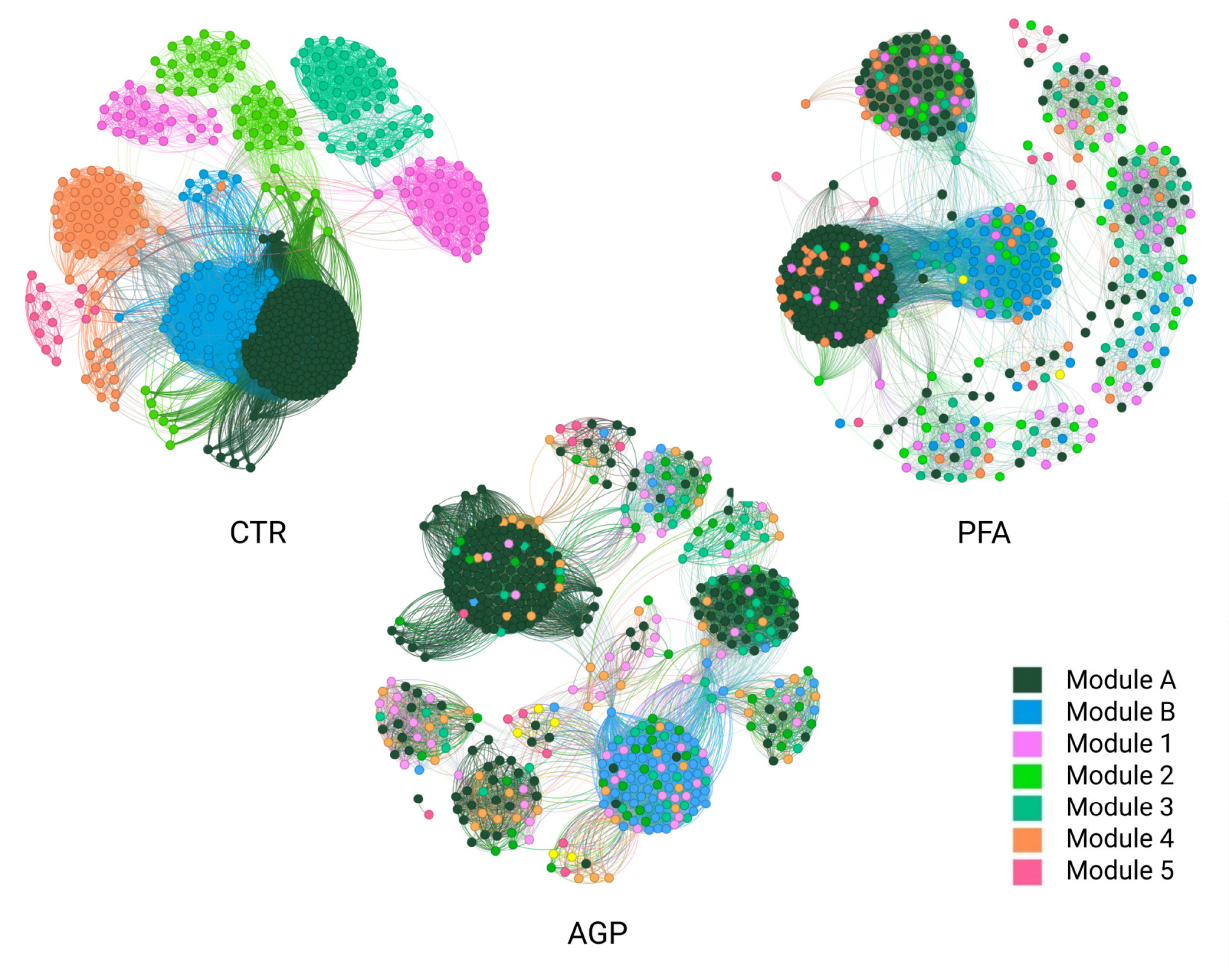
Module assignment of nodes in the constructed microbial co-occurrence networks at day 35. The nodes in the PFA and AGP networks are colored according to their module assignment in the CTR network.

Since the feed additives appeared to increase microbial network modularity, we further investigated the potential network modular organization shifts during the experiment. Our analysis showed that although the microbial networks had a distinct overall modular structure, two specific modules (labeled module *A* and module *B*) were consistently conserved across treatment groups and experimental time. Figure 2 illustrates the modular structure of the networks at day 35, with nodes in the AGP and PFA networks colored to match the Control network modules for easier comparison. Supplementary Figure S3 provides similar results for earlier time points.

Interestingly, module *A* exhibited a notable difference between groups: in the AGP and PFA networks, this module tends to split into two separate subgroups, each associated with different sample types – mucosa and digesta (Supplementary Figure S4). In contrast, in the Control networks, species in module *A* were primarily connected only to the digesta sample type. Additionally, such an effect is most notable on day 14 for both feed additive groups. Tracing the taxonomic lineages revealed that the majority of these species, uniquely associated with mucosa at this time point (27/28 for AGP and 14/16 in PFA) belong to the phyla *Firmicutes_A*. At the order level, 16 of these species were classified under *Lachnospirales* and 8 under *Oscillospirales* in the AGP group, whereas the PFA group had 6 such species under each of the two orders. Specifically, the majority of such *Lachnospirales* are further classied as *Lachnospiraceae*) in both feed additive groups (15/16 in AGP and 5/6 in PFA). This suggests that supplementation with feed additives promotes the colonization or association of species, especially *Lachnospirales* and *Oscillospirales*, with the mucosal region of the chicken cecum, a potentially important site for microbial-host interactions.

### 3.4 A statistically significant core structure exists across the constructed networks

To identify the conserved edges regardless of the treatment, we utilized anuran (Rttjers et al., 2021), a null-model-based method, to find the statistically significant core structure shared among networks built at day 14, 21 and 35. Specifically, anuran identified 1,684 edges that were shared in most of the networks (at least seven out of the nine networks). These edges involved a total of 127 species. *Anuran* confirmed the statistical significance of the identified core structure by comparing the results with the ones from random networks. In detail, the networks that preserved the original degree distribution had only 130 intersecting edges, while completely random networks, with no constraints, had just 4.

As presented in Table 2, this core consists of species belonging to various genera, with the most frequent ones being *Eisenbergiella, Mediterraneibacter, Blautia, Blautia_A* and *Flavonifractor*. The phyla composition indicates that the species are predominantly *Firmicutes_A* (87.4%), followed by *Firmicutes* (4.7%), *Actinobacteriota* (4.7%), *Bacteroidota* (2.4%), and *Proteobacteria* (0.8%).

**Table 2:** Species identified in the core structure categorized by the most frequent genus.

Genus	Species in the core structure
Eisenbergiella	Eisenbergiella intestinigallinarum, Eisenbergiella intestinipullorum, Eisenbergiella merdavium, Eisenbergiella merdigallinarum, Eisenbergiella pullistercoris, Eisenbergiella sp900555195, Eisenbergiella sp904392525, Eisenbergiella stercorigallinarum
Mediterraneibacter	Mediterraneibacter cottocaccae, Mediterraneibacter excrementigallinarum_A, Mediterraneibacter faecipullorum, Mediterraneibacter glycyrrhizinilyticus_A, Mediterraneibacter intestinigallinarum, Mediterraneibacter sp900541505, Mediterraneibacter sp900761655, Mediterraneibacter vanvlietii
Blautia	Blautia merdavium, Blautia ornithocaccae, Blautia pullistercoris, Blautia stercoravium, Blautia stercorigallinarum
Blautia_A	Blautia_A avistercoris, Blautia_A excrementigallinarum, Blautia_A gallistercoris, Blautia_A intestinipullorum
Flavonifractor	Flavonifractor avistercoris, Flavonifractor intestinigallinarum, Flavonifractor sp017811815

A full list of the species identified in the core structure can be found in Supplementary Table 2.

In general, these results support the existence of a stable core microbiome in the chicken cecum that appears consistently during chicken development even under different dietary regimens.

### 3.5 Detectable but limited shifts in keystone species composition following AGP supplementation

To investigate how key microbial species differ across diets, we first prioritized keystone taxa, species that play central roles in microbial networks, for each feeding group at three experiment time points. These taxa were selected from the influential nodes identified in the network analysis. We then examined the convergence and divergence of key taxa at these experiment phases and dietary conditions. Furthermore, we confirmed that at the baseline time, keystone taxa were very similar between the groups (Supplementary Figure S5).

**Figure 3:**
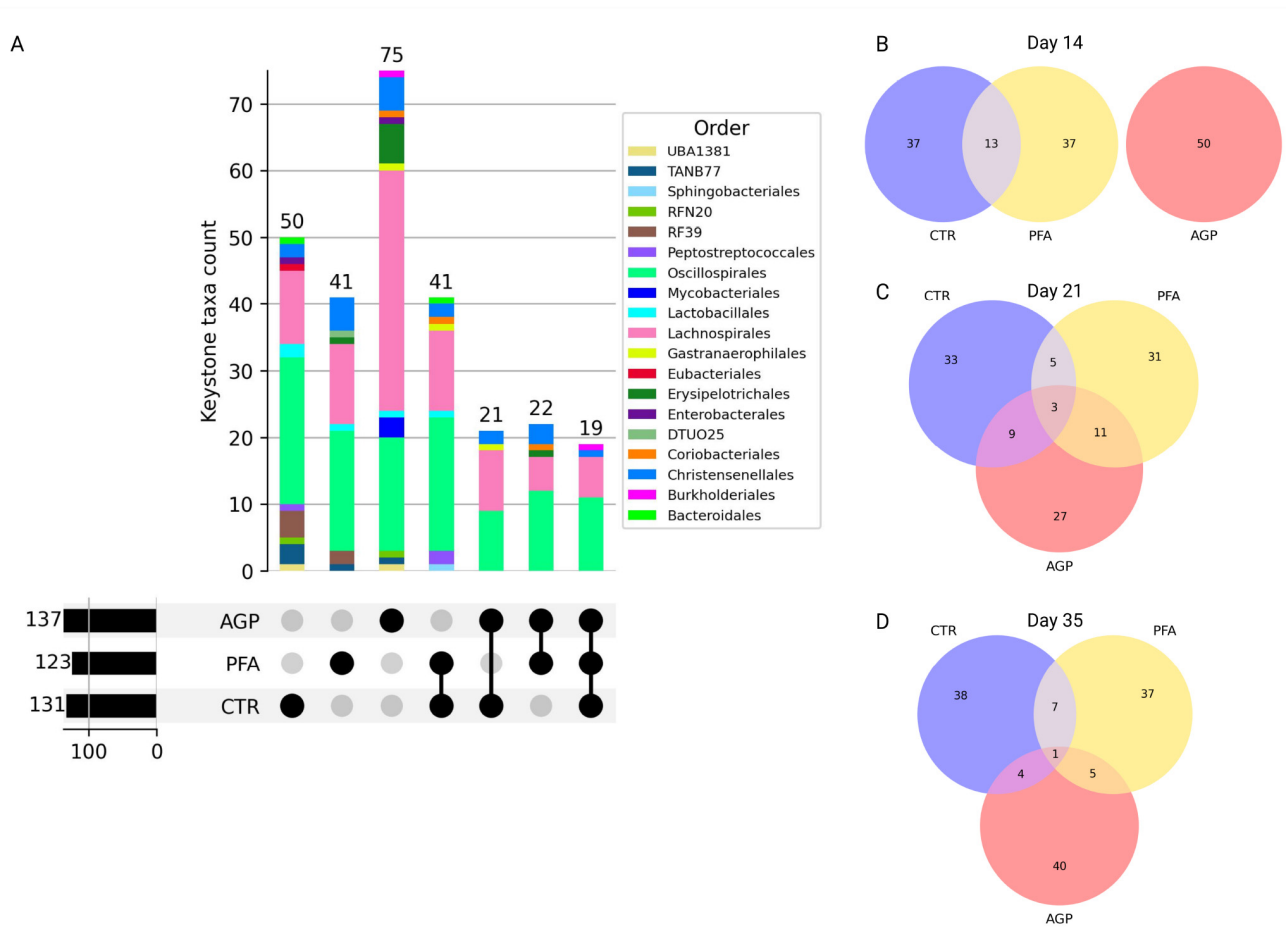
The overlapping and unique keystone taxa across the three treatment groups over the three developmental age during experiment. At each time point, the top 50 most influential nodes from the network constructed under each dietary condition were identified by node centrality analysis as keystone taxa. The shared and unique number of species between the three treatment groups at day 14, 21 and 35 are showed in **(B–D)**, respectively. These keystone taxa from the three time points were merged to form a single list for each group. The upset plot in **(A)** shows the number and the order of the merged keystone taxa unique to each treatment or shared between the treatments.

Throughout the experiment, the feed additives appear to increasingly alter the keystone taxa. At day 14, the CTR and PFA groups still shared a substantial number of keystone taxa, whereas AGP group showed a completely distinct profile (Figure 3B), suggesting a rapid response of the cecum microbiome to AGP. Notably, at this time point, approximately half of the keystone species belong to the order *Oscillospirales* in the CTR (27/50) and PFA (23/50) groups, which is higher than the AGP group (9/50). Conversely, the AGP group exhibits a notably higher proportion of keystone species from the order *Lachnospirales* (32/50) than the CTR (10/50) and PFA groups (14/50). Interestingly, by day 21, the keystone taxa became more similar between the PFA and AGP groups, while CTR and PFA shared less similarity compared to the previous time point (Figure 3C). On day 35, the three groups had developed relatively distinct keystone taxa profiles (Figure 3D). The detailed list of keystone species at each time can be found in the Supplementary Table 3.

To provide an overview of the unique and shared keystone taxa under the dietary conditions, we compiled a merged list of 269 keystone species identified over three experimental time and visualize their distribution between dietary groups (Figure 3A). Notably, AGP has the highest number of unique keystone taxa (75), followed by CTR (50) and PFA (41). Overall, the PFA and CTR groups share more keystone taxa with each other than either did with the AGP group. In total, nineteen species were shared between the three groups. The full list of species for each intersection is provided in the Supplementary Table 4.

Next, we assessed whether the abundance of these keystone taxa could predict the dietary group of each sample collected during the experiment. Using their abundance profile alongside developmental age, we perform supervised classification using Linear Discriminant Analysis (LDA) and Random Forest (RF) classifiers to classify dietary conditions, benchmarked against a stratified dummy classifier. Feature selection and hyperparameter tuning were embedded within a nested cross-validation framework to prevent information leakage and evaluate model performance. As a result, LDA significantly outperformed the dummy baseline for both accuracy and macro F1, while RF showed a significant improvement over the baseline only in accuracy (Supplementary Figure S6). However, overall predictive performance was modest (LDA: accuracy = 0.44 ± 0.13, macro F1 = 0.43 ± 0.13; RF: accuracy = 0.41 ± 0.128, macro F1 = 0.39 ± 0.12), suggesting that these keystone taxa abundances contain detectable but limited treatment-related information.

**Figure 4:**
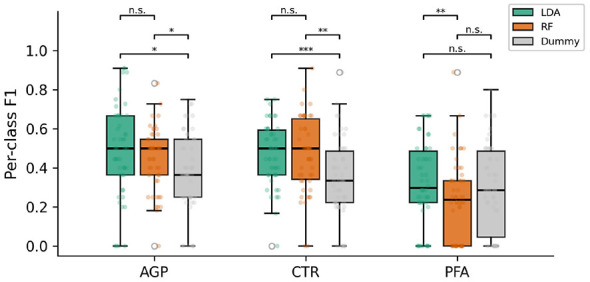
Per-class F1 scores of LDA, RF and dummy classifiers with pairwise significance tests across nested cross-validation. The distribution of achieved per-class F1 scores across 50 outer folds for the Linear Discriminant Analysis (LDA), Random Forest (RF), and dummy classifier. Brackets indicate two-sided paired *t*-test results (^***^p < 0.001, ^**^p < 0.01, ^*^p < 0.05, n.s., not significant).

Regarding per-class F1 scores, LDA significantly outperformed the dummy classifier for both AGP and CTR samples, while RF achieved significance only for CTR samples. Neither showed a significant improvement over the dummy classifier for PFA samples (Figure 4). The confusion matrix further illustrates the classification pattern (Supplementary Figure S7). For instance, LDA correctly classified approximately 51% of AGP and 49% of CTR samples per fold, compared with only 33% for PFA samples. Moreover, PFA misclassifications were distributed nearly equally between AGP (34%) and CTR (34%), suggesting that PFA samples lack a distinctive compositional signature. Together, these results indicate that AGP induces a more distinguishable shift in keystone species composition than PFA.

## 4 Discussion

Using a comparative network-based approach, we investigated how a commonly used antibiotic growth promoter (AGP) and a phytogenic feed additive (PFA) affect cecum microbial dynamics in broiler chicken. Compared to the Control group, both feed additives led to sparser microbial co-occurrence networks, with consistently increased modularity and a higher proportion of positive microbial associations. These findings indicate that although microbial richness remained largely unchanged, the internal organization and interaction balance of the community were substantially reshaped by dietary supplementation. The observed increase in network sparsity and modularity indicates a more compartmentalized network structure, with microbial species forming tightly connected subgroups with fewer interactions between them. We hypothesize that these subgroups represent ecologically coherent microbial guilds that have been restructured in response to selective pressure introduced by the additives. Assessing functional coherence within these guilds will require additional functional and metabolic analyses and therefore represents an important direction for future research. The sustained predominance of positive associations in the supplemented groups further suggests that feed additives may promote more cooperative microbial relationships, while the Control community progressively shifted toward a higher proportion of competitive interactions. Notably, the PFA induced changes that were directionally similar to those observed under AGP supplementation, although generally less pronounced. This suggests that phytogenic additives may partially recapitulate the ecological effects of antibiotic growth promoters while potentially exerting milder selective pressure.

Previous studies have shown that antibiotics and feed additives can substantially rewire the organization of microbial co-occurrence networks in livestock gut microbiota (Zou et al., 2022; Huang et al., 2018). However, systematic comparisons that examine detailed network topological characteristics remain limited. To our knowledge, this is the first study to report a consistent increase in modularity and a sustained high proportion of positive microbial edges in livestock gut microbial network associated with in-feed AGP and its natural alternative at multiple developmental stages. Interestingly, reduced network connectivity has been observed under antibiotic exposure in ruminants (Ji et al., 2018), suggesting a potentially conserved microbial response to such interventions across livestock species. These network-level findings complement our earlier analysis of the same dataset, which did not show significant differences in conventional community complexity metrics, including Shannon diversity, Pielou's evenness, and Chao1 richness, between the experimental groups (Peng et al., 2024). Similar observation made by Kumar et al. (2018), who also found minimal changes in taxonomic diversity in chicken gut microbiota following antibiotic treatment. Taken together, our results highlight the importance of examining community-level interaction patterns when evaluating microbiome shifts. This network-centric perspective is gaining traction in human microbiome research as well, with recent studies using similar approaches to gain insights into inflammatory bowel disease (IBD) (Baldassano and Bassett, 2016b) and colorectal cancer (CRC) (Xu et al., 2022). Collectively, these new findings suggest that topological organization of microbial networks may serve as a more informative biomarker of ecosystem perturbation than traditional diversity indices.

Building upon these insights, we further investigated the robustness of the microbial communities by simulating targeted node removal experiments. During the removal of influential taxa, both the AGP and PFA groups demonstrated enhanced network robustness, exhibiting a slower decline in connectivity and natural network integrity compared to controls. This increased robustness likely stems from a more balanced node degree distribution within the supplemented groups, suggesting that no single species dominates network connectivity excessively. Such a configuration may confer greater functional redundancy, allowing the community to better withstand disturbances. As Kajihara and Hynson (2024) highlighted, network robustness is tightly linked to the stability of complex biological systems, implying that feed additives could contribute to a more resilient gut ecosystem by buffering against perturbations that target at key microbial players. Future studies applying real-world stressors, such as pathogen challenges or dietary shifts, will be crucial to experimentally validate these in-silico predictions.

To further elucidate the ecological architecture of these microbial communities, we identified conserved modules present across all constructed networks during the experiment, regardless of age or treatment. Two dominant modules emerged consistently, confirmed through anuran (Rttjers et al., 2021) framework to represent shared association structures. The constituent taxa within these modules include genera like *Flavonifractor, Blautia, Eisenbergiella, Lachnoclostridium*, and *Mediterraneibacter*, which literature associates with beneficial functions in cecum ecosystem. For instance, *Flavonifractor* and *Blautia* correlate with improved feed conversion efficiency and growth performance in broiler chicken (Zhang et al., 2021), while *Eisenbergiella* and *Lachnoclostridium* contribute to butyrate production, a critical energy source for gut epithelial cells (Clavijo et al., 2022). Similarly, *Fournierella* appears to be implicated in maintaining poultry intestinal homeostasis (Liu et al., 2023), and *Mediterraneibacter* specializes in breaking down complex polysaccharides and plant fibers, generating metabolites beneficial to the host (Kim et al., 2019).

Our data further suggest that the feed additives may enhance the colonization of Firmicutes_A within these conserved modules, especially *Oscillospirales* and *Lachnospirales*. Additionally, most of such *Lachnospirales* are *Lachnospiraceae*. In a previous work, Richards et al. (2019) revealed that *Lachnospiraceae* are more abundant in the mucosal region of the cecum, indicating their potential roles in mucosal interaction with the host. Our finding complements the existing studies by highlighting the effects of feed additives to promote colonization of these beneficial taxa on the mucosal surface of the cecum. Future work should delve deeper into the spatial and functional characterization of microbial taxa.

In addition to the conserved modules, we performed node centrality analysis to identify keystone taxa unique to each treatment. We first confirmed that the keystone taxa profiles were extremely similar across the groups at day 3, the baseline time. At day 14, keystone profiles of CTR and PFA still exhibited a significant overlap, where as the AGP group displayed a distinct keystone composition. Specifically, a substantially higher proportion of keystone species from the order *Lachnospirales* is observed in the AGP group, with a reduced proportion from the order *Oscillospirales*. Coupled with the implication from the prior study from Richards et al. (2019) on the role of *Lachnospirales*, our findings further imply that the used AGP not only promoted mucosa-colonization of *Lachnospirales* and *Oscillospirales*, but also selectively prioritized *Lachnospirales* as keystone species, with potentially enhanced mucosal interactions. As development progressed, overlap between PFA and CTR keystone taxa declined, suggesting that AGP exerts an early and pronounced effect on critical microbial species, while PFA's effect unfolds more gradually. Validation of the functional role of these keystone species remains imperative, especially since network-based inferences assume that the co-occurrence pattern reflects true ecological interactions, a premise requiring empirical confirmation (Kajihara and Hynson, 2024).

Finally, we tested whether keystone taxa abundance could serve as a biomarker to classify microbial community differences between different feeding treatments using Linear Discriminant Analysis (LDA) and Random Forest (RF) within a nested cross-validation framework. While both model significantly outperformed the dummy classifier, their overall discriminative power remained modest. Notably, per-class analysis revealed that the classification of PFA samples from the two other groups is particularly difficult. This suggests that shifts in the keystone taxa abundance alone do not fully capture the subtle but meaningful effects of feed additives on cecum microbial community structure. These results reinforces the notion that topological changes in microbial communities–such as increased modularity or altered network connectivity can occur without substantial shifts in species abundance. In this context, the struggle of abundance-based classifiers to differentiate dietary groups highlights the added value of network-level metrics-the functional or structural rewiring of microbial communities may be more responsive to feed additive interventions than compositional shifts. This perspective aligns with the emerging concept of microbiome metabolic modulators (MMM) or precision biotics, which are designed to influence microbial function and cross-feeding interactions rather than targeting specific microbial taxa (Bortoluzzi et al., 2025; Walsh et al., 2021; Yan et al., 2023).

In summary, our study reveals critical, previously overlooked alterations in microbial community topology and dynamics induced by feed additives in broiler chicken cecum. Both AGP and PFA increased the sparsity and modularity of the microbial network of the cecum, changes that resulted in enhanced network robustness to perturbation. These alterations likely reflect the diversification of the functional roles of the cecum microbiome, which contributes to a more stable and resilient intestinal ecosystem. Despite these dynamic shifts, a conserved core microbial structure persists across treatment and developmental stages, underscoring the fundamental microbial framework essential for cecum homeostasis. Also, the tested feed additives potentially promote mucosa species colonization and interactions with host. The limited discriminative power of the abundance of keystone taxa for the classification of treatment highlights the complexity of microbiome responses, emphasizing that subtle changes in community-wide interaction may have greater biological relevance than individual taxon abundance alone. These insights lay a foundation for future efforts aimed at optimizing feed additive strategies as sustainable alternatives to antibiotics in livestock production.

## Data Availability

The original contributions presented in the study are included in the article/Supplementary material, further inquiries can be directed to the corresponding author.
