# Supplementary figures and images for: Antibiotic growth promoter and phytogenic feed additive consistently alter microbial community structure in chicken cecum
Source: Front Microbiol. 2026 Jun 4;17:1702973. doi: 10.3389/fmicb.2026.1702973 (PMC13275662; doi:10.3389/fmicb.2026.1702973)

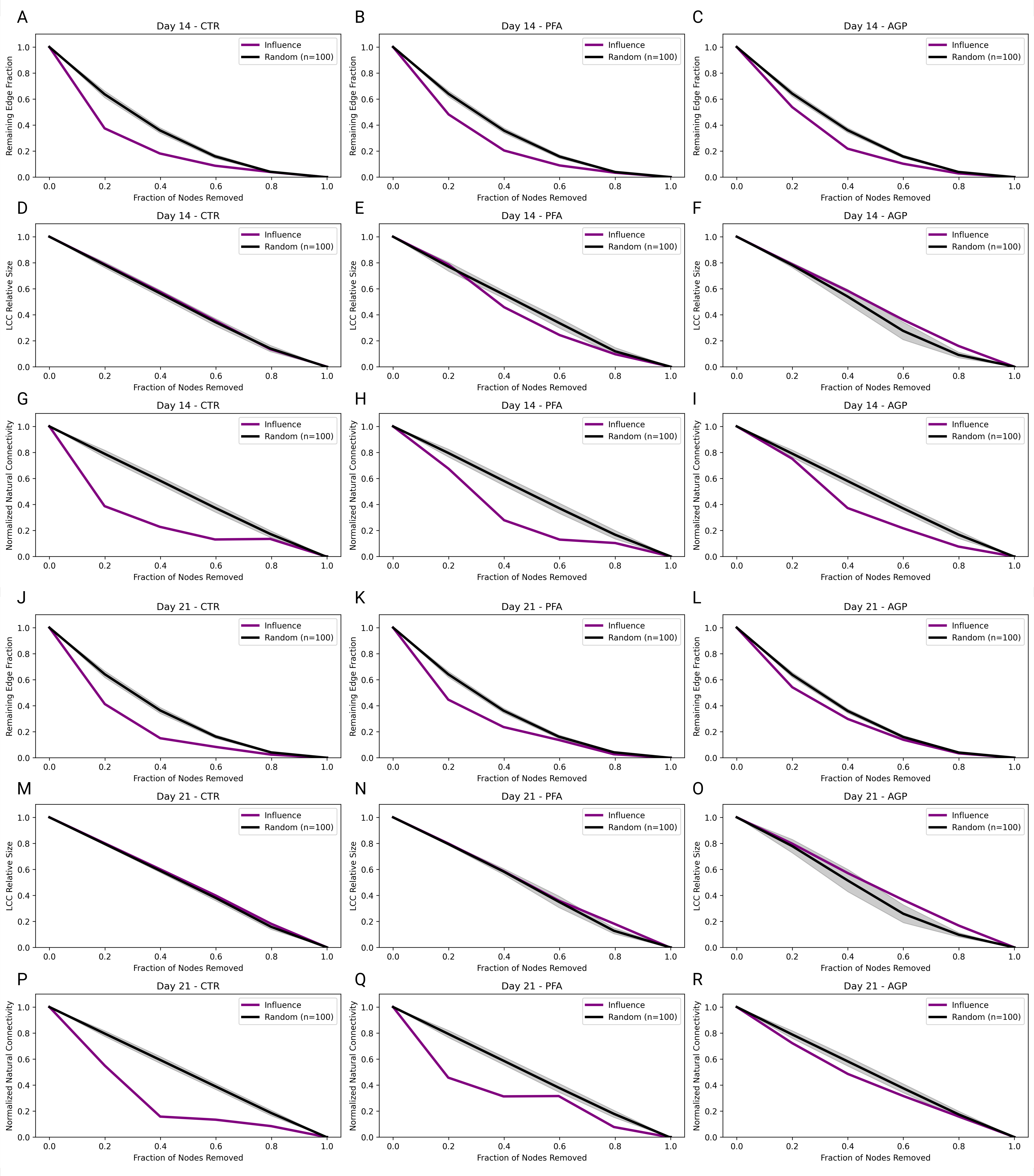

Supplement: Supplementary file 5 [file Image_1.jpeg]

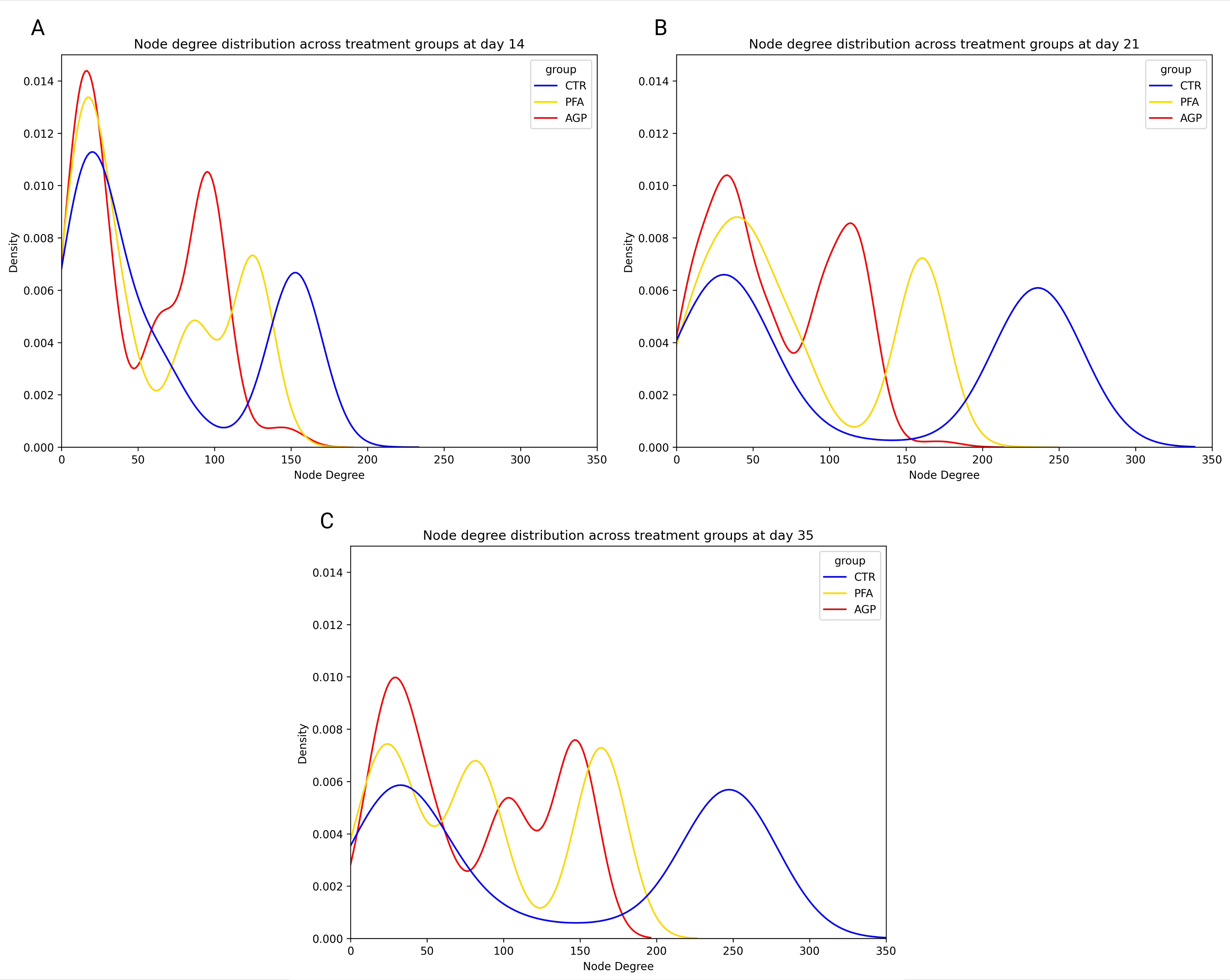

Supplement: Supplementary file 6 [file Image_2.jpeg]

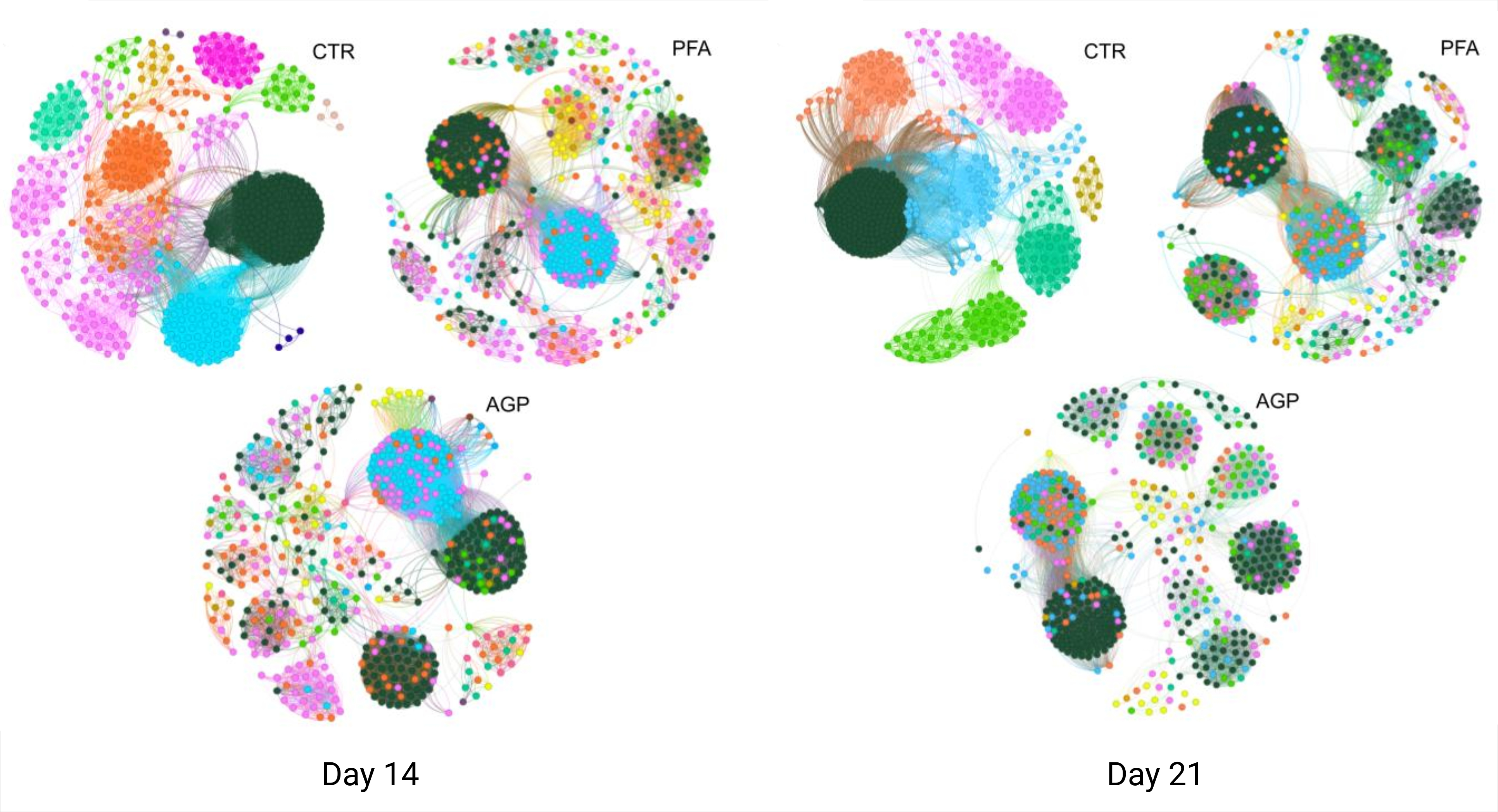

Supplement: Supplementary file 7 [file Image_3.jpeg]

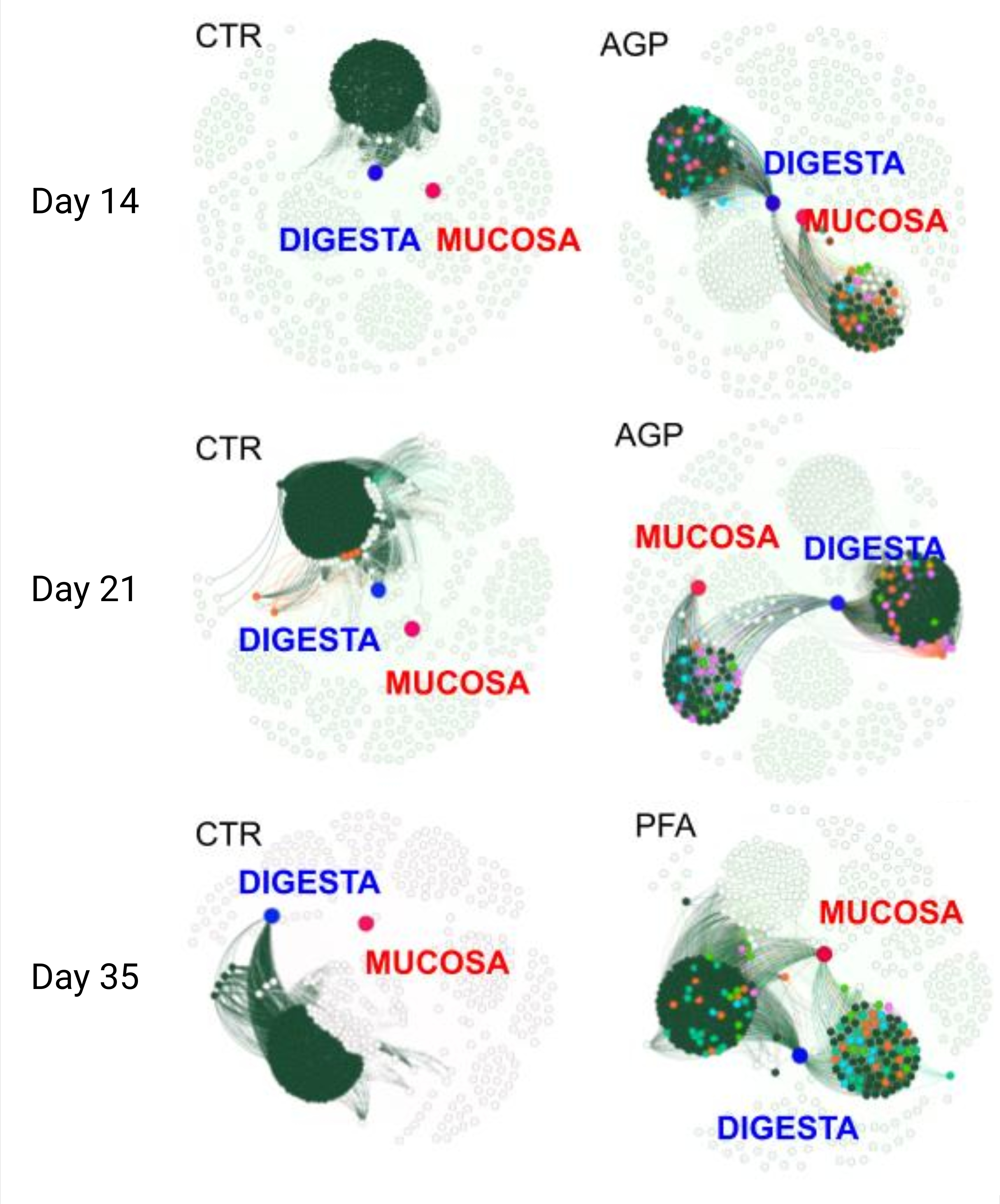

Supplement: Supplementary file 8 [file Image_4.jpeg]

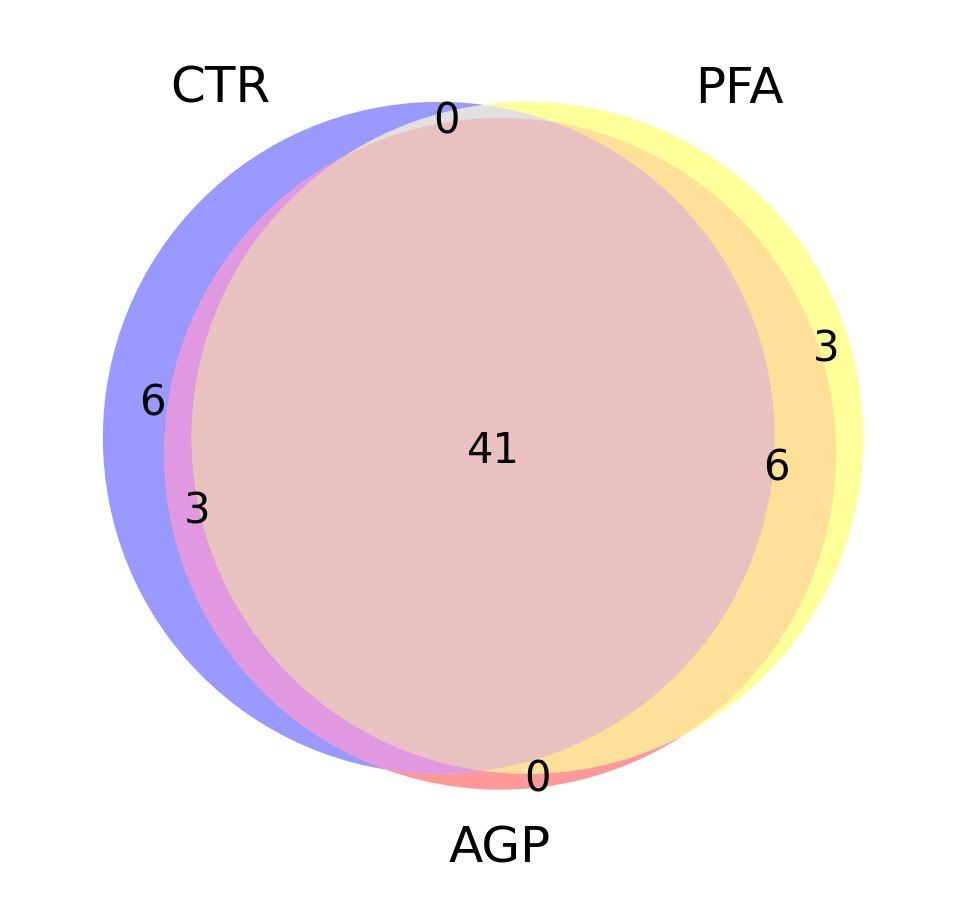

Supplement: Supplementary file 9 [file Image_5.jpeg]

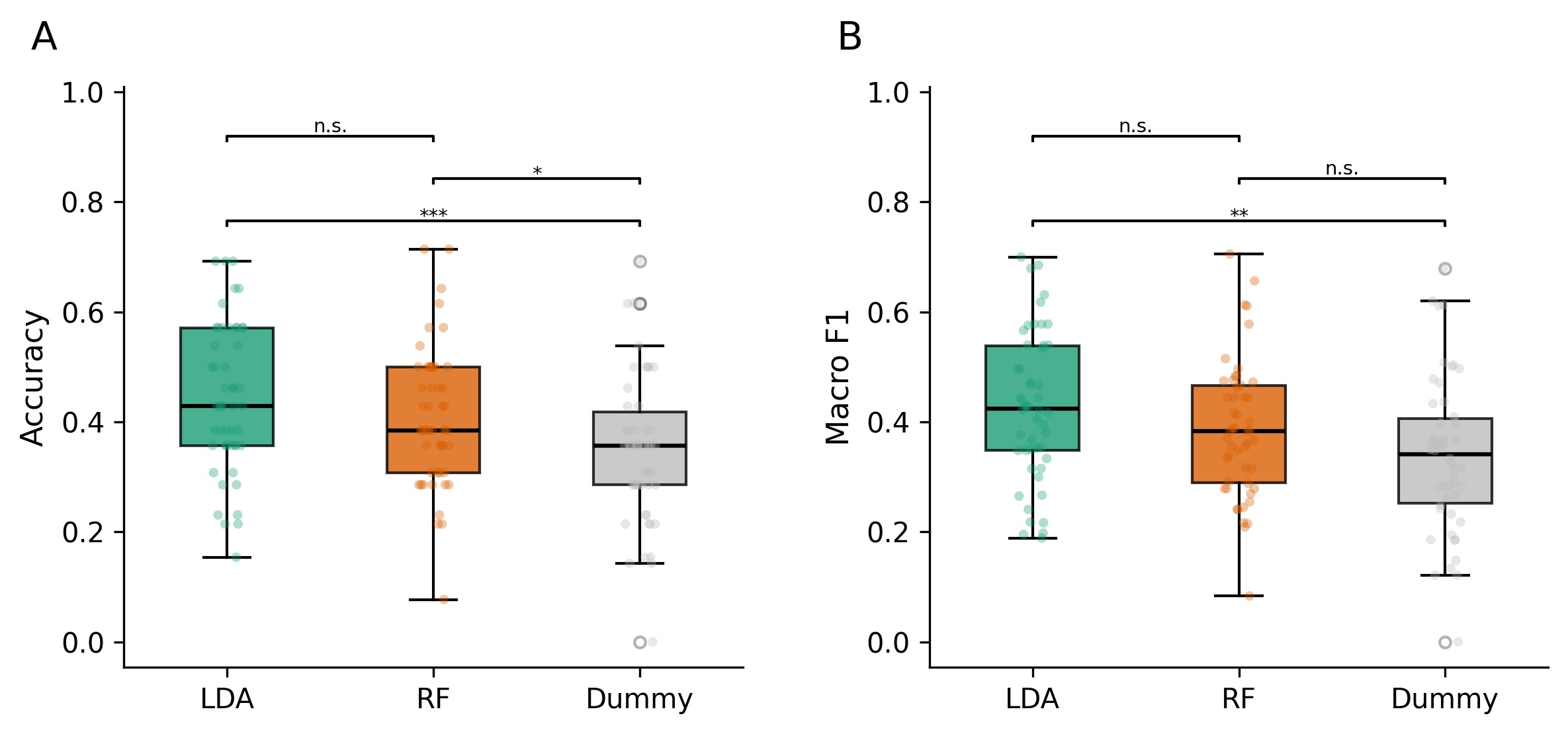

Supplement: Supplementary file 10 [file Image_6.jpeg]

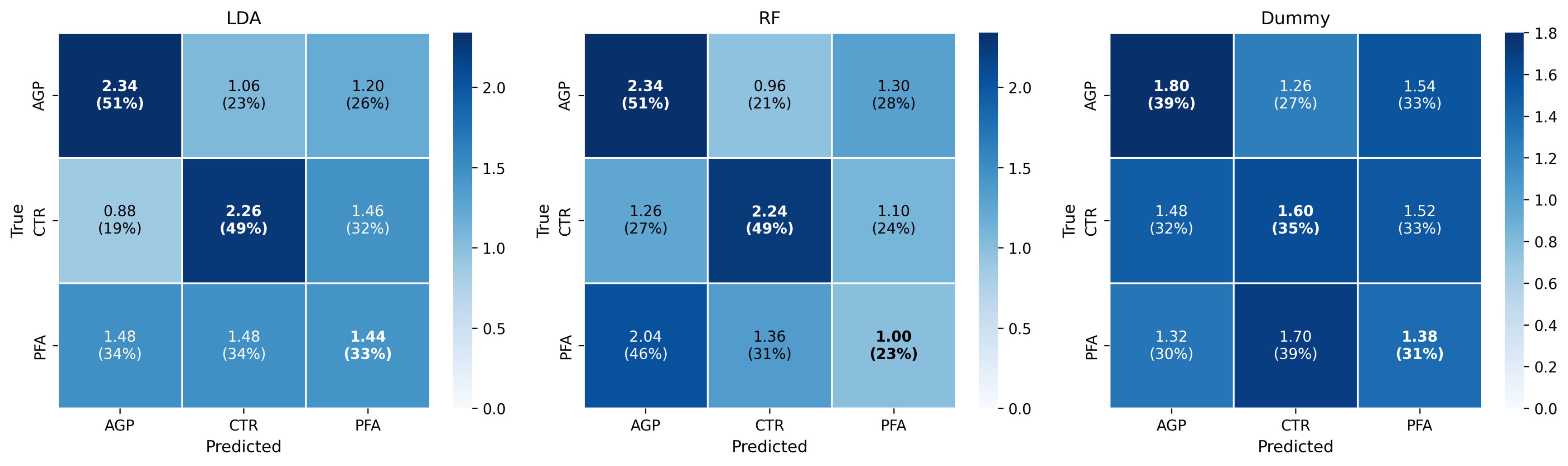

Supplement: Supplementary file 11 [file Image_7.jpeg]
